# PRF-Modified Graft Materials in Lateral Sinus Augmentation: A Systematic Review of Histologic Outcome

**DOI:** 10.3390/biomedicines14071593

**Published:** 2026-07-16

**Authors:** Guy Kukis, Gabriele Birbalaite, Audra Janovskiene, Žygimantas Petronis, Gabriele Sinkunaite, Dainius Razukevičius

**Affiliations:** 1 Faculty of Odontology, Medical Academy, Lithuanian University of Health Sciences, Eiveniu 2, LT-50161 Kaunas, Lithuania; guy.kukis@stud.lsmu.lt (G.K.); gabriele.birbalaite@stud.lsmu.lt (G.B.);; 2 Department of Maxillofacial Surgery, Medical Academy, Hospital of Lithuanian University of Health Sciences, Eiveniu 2, LT-50161 Kaunas, Lithuania; zygimantas.petronis@lsmu.lt (Ž.P.);

**Keywords:** platelet-rich fibrin, PRF, maxillary sinus augmentation, sinus lift, histomorphometry, bone regeneration, xenograft, allograft, DBBM, lateral window technique

## Abstract

**Background**: Platelet-rich fibrin (PRF) has been increasingly investigated as a biological adjunct in maxillary sinus augmentation procedures because of its potential to enhance bone regeneration and graft remodelling. However, the histologic benefits of combining PRF with allograft or xenograft materials during lateral sinus augmentation remain unclear. This systematic review aimed to evaluate the histomorphometric outcomes of PRF-modified graft materials compared with graft materials alone in lateral maxillary sinus augmentation. **Methods**: This systematic review was conducted according to PRISMA 2020 guidelines and registered in PROSPERO (CRD420261301066). Electronic searches were performed in PubMed, Cochrane Library, and Google Scholar databases for studies published within the last 10 years. Randomized controlled trials and split-mouth randomized studies evaluating histologic and histomorphometric outcomes following lateral sinus augmentation using allograft or xenograft materials with and without PRF were included. A random-effects meta-analysis was conducted, and effect estimates were expressed as Hedges’ g and presented with corresponding 95% confidence intervals (CIs). **Results**: Four randomized clinical studies involving lateral sinus augmentation procedures were included. Histomorphometric findings generally demonstrated higher percentages of newly formed bone and lower residual graft material in PRF-treated groups compared with graft materials alone. Meta-analyses showed a significant increase in newly formed bone, favouring PRF (effect size 0.61; 95% CI: 0.15 to 1.08; *p* = 0.01), and a significant reduction in residual graft material (effect size −0.65; 95% CI: −1.15 to −0.14; *p* = 0.01). No statistically significant differences were observed regarding connective tissue/marrow spaces or radiographic bone height. Heterogeneity among studies was low to moderate. **Conclusions**: The adjunctive use of PRF during lateral maxillary sinus augmentation appears to enhance early bone regeneration and graft remodelling by increasing newly formed bone and reducing residual graft particles. However, radiographic bone dimensions were generally comparable between PRF-treated and control groups. PRF may therefore be considered a promising biological adjunct rather than a substitute for conventional grafting materials. Further standardized randomized clinical trials with larger sample sizes are required to clarify its long-term clinical effectiveness.

## 1. Introduction

Alveolar bone resorption is an unavoidable sequel of tooth extraction, leading to inadequate bone mass. In addition, maxillary sinus pneumatization limits adequate vertical ridge dimensions in the posterior maxilla. Thus, maxillary sinus floor elevation procedures have gained great interest over the past decade to overcome these drawbacks [1]. This procedure enables the reconstruction of atrophic maxillary ridges with an implant-supported prosthesis.

Since its first introduction in the 1960s, sinus floor augmentation has been a well-established surgical procedure that has undergone numerous revisions. A major milestone was reported in 1980 when Boyen and James successfully used this treatment method in a patient with severe maxillary sinus pneumatization to facilitate implant rehabilitation of the posterior maxilla [2,3].

To date, various sinus augmentation approaches have been reported utilizing autogenous bone, xenografts, and/or allografts. The most well-documented technique is the lateral window approach [4].

Despite satisfactory clinical outcomes utilizing various biomaterials, including allografts and xenografts during maxillary sinus augmentation (MSA), there is still great debate regarding the most effective grafting combinations and clinical conditions for maximizing bone regeneration and maintaining long-term volume stability [5].

The selection of grafting materials for MSA is primarily based on their osteoconductive, osteoinductive, and osteogenic properties [6,7,8].

During the lateral window technique, elevation of the Schneiderian membrane creates a compartment that is subsequently filled with graft material. Bone regeneration is initiated by the migration of osteogenic precursor cells; thus, osteogenesis commences from the periphery and continues toward the central and apical areas [9].

Due to its osteogenic and osteoinductive potential, autogenous bone is considered the reference grafting material and is associated with new bone formation during the early healing phase; nonetheless, it has been demonstrated that they typically resorb rapidly [7].

Alternative grafting materials have therefore been investigated to enhance graft stability, such as deproteinized bovine bone mineral (DBBM), which is a porous carbonate apatite crystal and is derived from the calf bone. They have a high osteoconductive activity, osteoblastic differentiation, and extracellular bone matrix deposition [10]. However, they have low bone regeneration capacity [11]. Hence, combining the graft material in combination with a biologic enhancer that contains vital growth factors involved in bone formation patterns may shorten the time it takes for the graft to heal and enhance the osteoinductive process [12].

Platelet-rich fibrin (PRF), a second-generation platelet-rich concentration, was created in 2000 by Choukroun et al. in France [13]. PRF is completely autologous and straightforward regarding preparation, as it simply consists of centrifuged blood, which has prolonged growth factor release. Owing to its high concentration of bioactive molecules, PRF has been associated with enhanced angiogenesis, cell proliferation, tissue remodelling, and bone regeneration [14,15].

PRF exerts its regenerative effects through several interconnected biological mechanisms. Its three-dimensional fibrin scaffold enables the sustained release of platelet and leukocyte-derived growth factors, including platelet-derived growth factors (PDGFs), transforming growth factor-β (TGF-β), insulin-like growth factor-1 (IGF-1), and vascular endothelial growth factor (VEGF) [16]. PDGF and TGF-β recruit mesenchymal stem cells and promote their differentiation into osteoblasts, while VEGF drives angiogenesis by stimulating endothelial cell proliferation [17]. PRF further upregulates osteogenic markers such as BMP-2 and Runx2, supporting matrix mineralisation, while its leukocyte content contributes anti-inflammatory activity that favours a stable healing environment [18].

Previous systematic reviews have evaluated PRF in maxillary sinus augmentation, but each has notable scope limitations. Earlier reviews predate the introduction of several PRF subtypes and were largely restricted to L-PRF alone [19]. More recently, Almutairi et al. (2025) conducted a meta-analysis restricted specifically to L-PRF combined with DBBM, excluding allograft comparisons and other PRF preparations such as H-PRF and T-PRF [20]. The present review addresses this gap by pooling histomorphometric outcomes across both allograft and xenograft comparisons and by including multiple contemporary PRF protocols (L-PRF, H-PRF, and T-PRF), thereby providing a more comprehensive synthesis of the currently available randomized evidence on PRF-modified graft materials in lateral sinus augmentation.

Although PRF has been extensively investigated as an adjunctive biomaterial in MSA, evidence directly comparing its use in combination with allografts and xenografts remains limited.

Hence, the purpose of this study was to compare the histomorphometric outcomes of the regenerated vital bone and volume of new bone in the grafted maxillary sinus using allografts with and without PRF and xenografts with and without PRF.

## 2. Materials and Methods

This systematic review was conducted in accordance with the Preferred Reporting Items for Systematic Reviews and Meta-Analyses (PRISMA) guidelines (PRISMA checklist shown in Appendix A) [21]. A main question for the study was as follows: In patients undergoing lateral maxillary sinus augmentation, does the use of PRF combined with allograft or xenograft materials, compared with graft materials alone, improve the histomorphometric outcomes of bone regeneration and volume of new bone tissue? The following PICO strategy was established.


The population(P)Patients presenting with atrophic posterior maxilla who underwent lateral maxillary sinus augmentation prior to dental implant placement.The intervention(I)Lateral sinus augmentation performed using allografts with PRF and xenografts with PRF.The control(C)Lateral sinus augmentation performed using the same graft materials without PRF, including allograft alone and xenograft (DBBM) alone.The outcomes(O)Primary Results: histomorphometric and/or histologic parameters of bone regeneration, including percentage of newly formed bone, residual graft material, and qualitative bone characteristics. Secondary Results: radiographic parameters including bone height gain, graft volume, and bone density.


The protocol for this systematic review and meta-analysis was registered with PROSPERO: CRD420261301066.

### 2.1. Search Strategy

**Figure 1:**
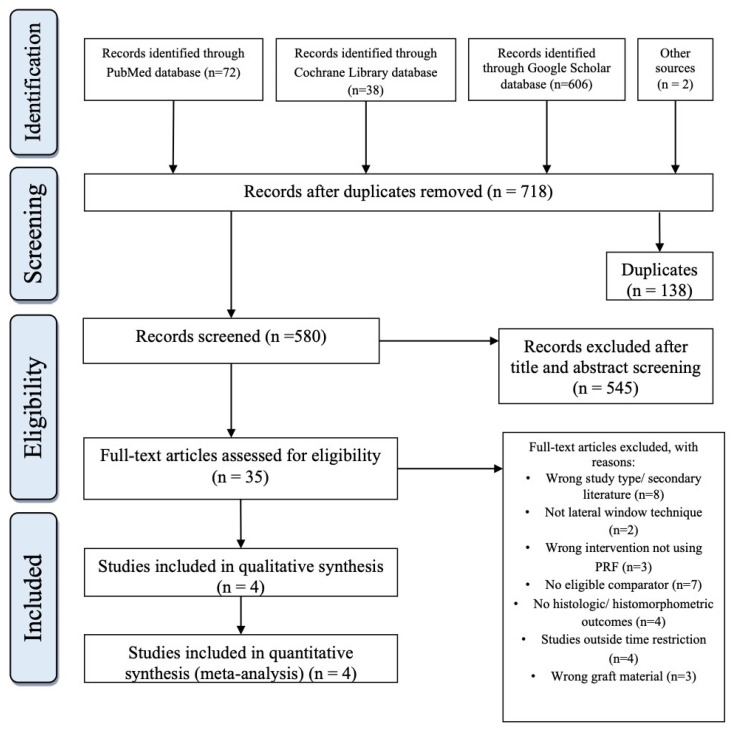
PRISMA flow chart of included searches and screening.

An electronic search was conducted independently and in duplicate by two reviewers (G.K and G.B) in March 2026. The search was carried out in the PubMed, Cochrane Library, and Google Scholar databases using an identical Boolean search string across all three databases. For Google Scholar, all retrieved records matching the search string were screened by title, consistent with the number of records reported in Figure 1.

The following keywords and Boolean operators were used in the search strategy: (‘platelet-rich fibrin’ OR ‘PRF’ OR ‘L-PRF’ OR ‘H-PRF’ OR ‘T-PRF’) AND (‘sinus augmentation’ OR ‘lateral window sinus augmentation’) AND (‘histomorphometry’ OR ‘histology’ OR ‘bone regeneration’ OR ‘bone volume’) AND (‘bone graft’ OR ‘allograft’ OR ‘xenograft’ OR ‘DBBM’).

The literature search was restricted to articles written in the English language and published within the past 10 years. No search limitations regarding the country’s publication or status were applied.

The selection of publications was carried out in two stages. In the first stage, duplicate records were removed, and titles and abstracts were screened for relevance according to the predefined eligibility criteria. Studies that did not meet the inclusion criteria were excluded. In the second stage, the full texts of potentially eligible articles were assessed and included or excluded based on the established inclusion and exclusion criteria. In addition, the reference list of the included studies was manually screened to identify any additional relevant publications.

Title and abstract screening, full-text eligibility assessment, and data extraction were likewise performed independently and in duplicate by the same two reviewers (G.K and G.B). No disagreements occurred between the reviewers at any stage of study selection or data extraction; therefore, no third-party adjudication was required.

### 2.2. Selection Criteria

Inclusion criteria encompass randomized controlled trials, as well as observational, retrospective studies, single-blinded, split-mouth randomized studies, and controlled clinical trials. Eligible studies directly compared lateral maxillary sinus augmentation performed using bone graft materials alone versus graft materials combined with PRF and reported histologic and/or histomorphometric outcomes of bone regeneration and volume.

### 2.3. Exclusion Criteria

Studies were excluded if they were

- Animal or in vitro studies, case reports, narrative reviews, or meta-analyses;
- Studies using transalveolar (crestal) sinus lift techniques only;
- Studies lacking histologic or histomorphometric evaluation;
- Studies evaluating PRF without a bone graft material;
- Studies evaluating graft materials other than allografts or xenografts;
- Non-English publications or studies with unavailable full text.

### 2.4. Risk of Bias

The risk of bias of the included prospective randomized studies was assessed using ‘The Cochrane Collaboration’s risk-of-bias (RoB 2) tool’ questionnaire [21]. The RoB 2 tool evaluates five domains of potential bias, including the randomization process, deviations from intended interventions, missing outcome data, outcome measurement, and selection of reported results. Using these algorithms, each standardized criterion is assigned a risk rating: low (+), medium (−), and high (×).

### 2.5. Statistical Analysis

A meta-analysis was conducted to quantitatively synthesize the histomorphometric outcomes across the included studies. Continuous outcomes were analysed using Hedges’ g with 95% confidence intervals (CIs), applying a random-effects model [22,23].

Statistical heterogeneity among studies was assessed using Cochran’s Q test and quantified using the $I^{2}$ statistic. An $I^{2}$ value of 0–40% was considered low heterogeneity, 30–60% moderate, 50–90% substantial, and 75–100% considerable heterogeneity, as previously described by Higgins et al. [24].

A fixed-effects model was applied when heterogeneity was low and not statistically significant; otherwise, a random-effect model was considered. Statistical significance was set at *p* < 0.05.

Forest plots were generated to visually represent the pooled estimate of the effect size.

Given the small number of included studies (k = 4), formal assessment of publication bias was not performed.

## 3. Results

### 3.1. Study Selection

During the initial stage of the literature search, 718 publications were extracted based on the determined keyword combination. Of these publications, 138 were duplicates. Following duplicate removal, 580 records underwent title and abstract screening. Of these, 35 studies met the eligibility criteria and proceeded to full-text assessment. Applying the rejection criteria, 29 publications were excluded due to being of the wrong study type/secondary literature (k = 8), studies not using the lateral window technique (*n* = 2), studies using the wrong intervention (not using PRF) (*n* = 3), studies with no eligible comparator (*n* = 7), studies with no histologic or histomorphometric outcomes analysed (*n* = 4), studies outside of the 10-year time restriction (*n* = 4), and studies using the wrong graft material (*n* = 3). Four publications [25,26,27,28] were included in the systematic literature review. The search proceeding diagram is presented in Figure 1.

### 3.2. Quality Assessment of the Included Studies

The ‘The Cochrane Collaboration’s risk-of-bias (RoB 2) tool’ questionnaire was used to evaluate the risk of bias in prospective randomized studies [29]. Each of the five standardized criteria in the RoB 2 tool has an evaluation methodology. Each standardized criterion is given a risk rating using these algorithms: low (+), medium (−), and high (×).

### 3.3. Risk-of-Bias Assessment

**Figure 2:**
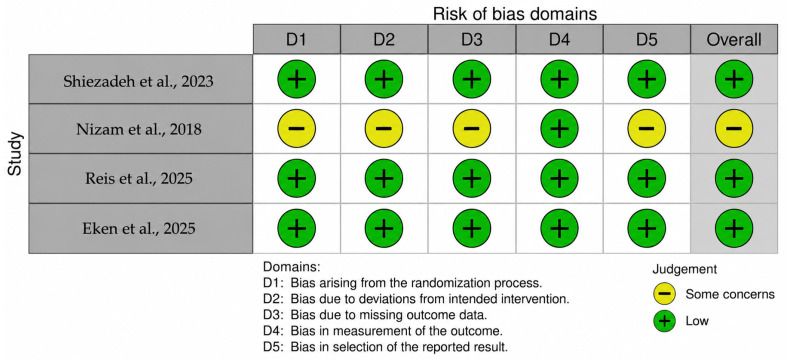
Risk-of-bias assessment of included studies in the review [25,26,27,28].

Once ‘The Cochrane Collaboration’s risk-of-bias (RoB 2) tool’ was used, it was concluded that overall, three studies [25,27,28] were judged to have a low risk of bias, while one study [26] was rated as having some concerns. In this study, potential methodological limitations were mainly related to incomplete reporting of the randomisation process and uncertainties regarding outcome reporting and measurement procedures. A visual summary (using the ‘Robvis’ tool) of the risk-of-bias assessment across the five RoB 2 domains is presented in Figure 2.

### 3.4. Characteristic of Included Studies

Four studies [25,26,27,28] were included in this literature review. The included study designs consisted of randomized controlled trials and split-mouth randomized studies. All included studies investigated at least one outcome matching the eligibility criteria: histologic and/or histomorphometric indicators of regenerated vital bone, including the percentage of newly formed bone, residual graft material, and/or marrow/connective tissue spaces, as well as, in some studies, radiographic assessment of bone volume or density changes in the grafted maxillary sinus.

Four of the included studies solely assessed histomorphometric percentage parameters, with a primary focus on newly formed vital bone and residual graft content following healing. In addition to microscopic results, all studies assessed radiographic (CBCT or conventional radiology) parameters and histomorphometric results, enabling evaluation of bone volume stability and changes in bone density.

Overall, the included evidence allowed direct comparisons of the regenerated bone outcomes in lateral maxillary sinus augmentation utilizing allografts with and without PRF, as well as xenografts with and without PRF. All extracted results are shown in Table 1.

### 3.5. Histomorphometric Bone Regeneration Outcomes

All four included randomized clinical trials evaluated histomorphometric indicators of bone regeneration after lateral maxillary sinus augmentation using graft materials with and without platelet-rich fibrin (PRF). The healing periods ranged from four to six months. They assessed the percentage of newly formed bone, residual graft percentage, and marrow generation and/or connective tissue percentage [25,26,27,28]. Values are presented as mean ± standard deviation (SD).

In the study by Reis et al. [27] in 2025, there was a follow-up of four months. Twenty-six histological sections were evaluated. The DBBM combined with the H-PRF group exhibited more newly produced bone (51.33% ± 6.17%) than in the DBBM alone group (45.68% ± 6.65%) (*p* < 0.05). The percentages of residual graft were 21.85% ± 8.93% and 24.09% ± 11.79%, and those of soft tissue were 26.81% ± 9.08% and 30.21% ± 9.06%. No statistically significant differences were observed between the groups for these parameters. Although the healing period was limited to four months, an immature bone matrix was identified in specimens from both treatment groups, with variability in its distribution and extent. Compared with DBBM alone, the DBBM group combined with H-PRF demonstrated increased new bone formation and a greater number of osteogenic areas. In addition, areas exhibiting active osteogenesis appeared more frequently and were distributed more extensively when H-PRF was used as an adjunct [27].

In the randomized clinical trial conducted by Shiezadeh et al. [25], twenty patients underwent lateral maxillary sinus augmentations with either PRF combined with an allograft (Group A) or an allograft alone (Group B). Upon a healing period of six months, histomorphometric analysis demonstrated that the percentage of newly formed bone was 43.25% ± 5.22% in the PRF-combined allograft group and 38.25% ± 7.01% in the allograft-only group. The proportion of residual graft material was lower in Group A (9.35% ± 3.43%) compared with Group B (13.18% ± 3.67%). Additionally, bone marrow generation was reduced in Group A (6.81% ± 2.19%) compared with Group B (10.23% ± 4.49%).

The split-mouth randomized clinical trial by Nizam et al. [26] included twenty-one of the twenty-six sinuses undergoing sinus augmentation with DBBM alone (control) and DBBM combined with L-PRF (test), which were suitable for the histomorphometric analysis. Histomorphometric evaluation performed after six months showed comparable percentages of newly formed bone between the groups, with 21.38% ± 8.78% in the L-PRF group and 21.25% ± 5.59% in the control group, which showed no significant difference. Regarding the residual bone graft material, the test group resulted in a lower percentage (25.95% ± 9.54%) compared to the control group (32.79% ± 5.89%). The connective tissue component showed slightly greater results (52.67% ± 12.53%) in the test group compared to the control group (45.96 ± 8.36%).

In the study by Eken et al. [28], ten patients underwent sinus augmentation using titanium-prepared PRF (T-PRF) or DBBM graft material. After six months of healing, histomorphometric analysis revealed greater new bone formation in the T-PRF group (19.48% ± 14.60%) than in the DBBM group (8.31% ± 5.47%), with the difference reaching statistical significance. The residual graft material was significantly greater in the DBBM group (4.81% ± 6.67%) than in the T-PRF group, where no residual graft particles were detected (0.00% ± 0.00%). The connective tissue component was similar between the groups, measuring at 21.60% ± 12.43% in the T-PRF group and 21.49% ± 11.77% in the DBBM group.

### 3.6. Clinical and Radiographic Findings

Reis et al. [27] conducted radiographic and micro-computed tomography (micro-CT) analyses, evaluating bone regeneration. Prior to sinus augmentation, the average residual bone height was 2.6 ± 0.6 mm in the DBBM group (control) and 2.1 ± 0.9 mm in the DBBM combined with H-PRF group (experimental). Baseline residual bone height values did not differ significantly between the treatment groups (*p* = 0.418). Micro-CT demonstrated significantly higher bone volume fractions in the experimental group (4485 ± 1469) compared with the control group (2562 ± 1271). Conversely, the material volume percentage representing remaining bone substitute particles was significantly higher in the DBBM-only group. Overall, these findings suggest that the addition of H-PRF to DBBM may enhance bone microarchitecture, as indicated by the increased bone volume fraction and connectivity density, while reducing the proportion of residual graft material.

Shiezadeh et al. [25] assessed the radiographic outcomes after a six-month healing period. Radiographic evaluation demonstrated comparable sinus bone dimensions between the two groups. The mean sinus bone width was 6.24 ± 1.07 mm in the PRF plus allograft group and 6.25 ± 1.27 mm in the allograft-only group. Similarly, the mean bone height measured 2.74 ± 0.88 mm in the PRF group and 2.72 ± 0.92 mm in the control group. No statistically significant differences were described between the groups regarding these radiographic parameters. These findings suggest that although the addition of PRF influenced certain histomorphometric parameters, including reduced residual graft particles and increased bone marrow formation, it did not significantly affect radiographic bone dimensions following maxillary sinus augmentation.

Nizam et al. [26] measured the radiographic outcomes, which were performed six months after sinus augmentation using DBBM alone or combined with L-PRF. Similar radiographic appearances were seen, demonstrating comparable augmented bone height between the two groups. The mean augmented sinus bone height measured 13.60 ± 1.09 mm in the DBBM combined with the L-PRF group and 13.53 ± 1.20 mm in the DBBM-only group. No statistically significant difference was observed. These results indicate that the addition of L-PRF to DBBM did not statistically influence radiographic bone height following sinus augmentation.

Eken et al. [28] evaluated radiographic bone changes after maxillary sinus augmentation using DBBM alone compared with DBBM combined with T-PRF. The residual bone height was 3.32 ± 1.26 mm in the T-PRF group and 2.78 ± 1.10 mm in the DBBM group, with no statistically significant difference. However, several radiographic parameters were significantly higher in the DBBM group. The total bone height measured at 10.64 ± 3.96 mm in the T-PRF group and 14.25 ± 1.65 mm in the DBBM group. Similarly, bone gain was 7.33 ± 3.75 mm in the T-PRF group compared with 11.47 ± 2.14 mm in the DBBM group. The graft volume was 989.89 ± 523.07 mm^3^ in the T-PRF group and 1519.39 ± 432.61 mm^3^ in the DBBM group. In addition, bone density values were 192.09 ± 127.90 HU in the T-PRF group and 492.77 ± 117.35 HU in the DBBM group, showing significantly greater density in the DBBM group.

### 3.7. Meta-Analysis Summary

#### 3.7.1. Newly Formed Bone

**Figure 3:**
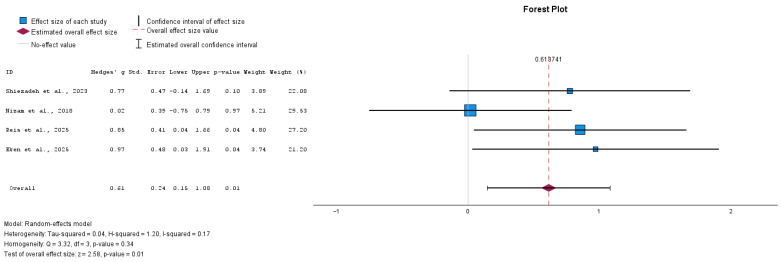
Forest plot of the effect of PRF on newly formed bone (%) following sinus augmentation [25,26,27,28].

Figure 3 illustrates the pooled effect of PRF on the percentage of newly formed bone. The overall effect size was 0.61 (95% CI: 0.15 to 1.08), demonstrating a statistically significant difference in favour of the PRF group (Z = 2.58, *p* = 0.01). The heterogeneity among studies was low ($I^{2}=17\%)$, indicating consistent findings across the included trials. These results suggest that the adjunctive use of PRF significantly enhances new bone formation during the healing period following sinus augmentation.

#### 3.7.2. Residual Graft Material

**Figure 4:**
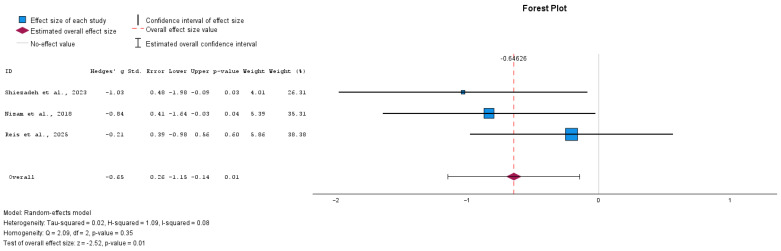
Forest plot of the effect of PRF on residual graft material (%) following sinus augmentation [25,26,27].

Figure 4 presents the pooled analysis of residual graft material. The overall effect size was −0.65 (95% CI: −1.15 to −0.14), showing a statistically significant reduction in residual graft content in the PRF group compared with controls (Z = −2.52, *p* = 0.01). Heterogeneity was low ($I^{2}=8\%)$, reflecting high consistency between studies. This finding indicates that PRF may promote more efficient graft remodelling and resorption of graft particles.

#### 3.7.3. Connective Tissue/Marrow Spaces

**Figure 5:**
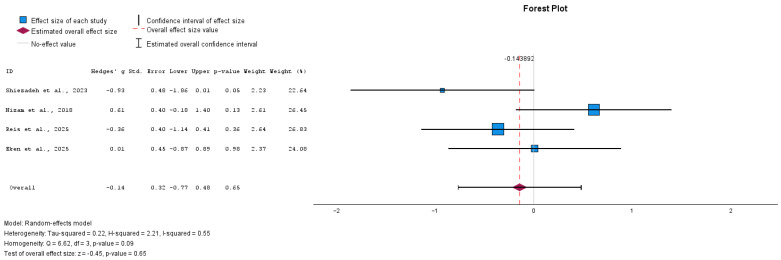
Forest plot of the effect of PRF on connective tissue and marrow spaces (%) following sinus augmentation [25,26,27,28].

Figure 5 demonstrates the pooled effect of PRF on connective tissue and marrow space formation. The overall effect size was −0.14 (95% CI: −0.77 to 0.48), with no statistically significant difference between groups (Z = −0.45, *p* = 0.65). Moderate heterogeneity was observed ($I^{2}=55\%)$, suggesting variability among study outcomes. These results indicate that PRF does not exert a consistent or significant influence on connective tissue or marrow space proportions.

#### 3.7.4. Bone Height

**Figure 6:**
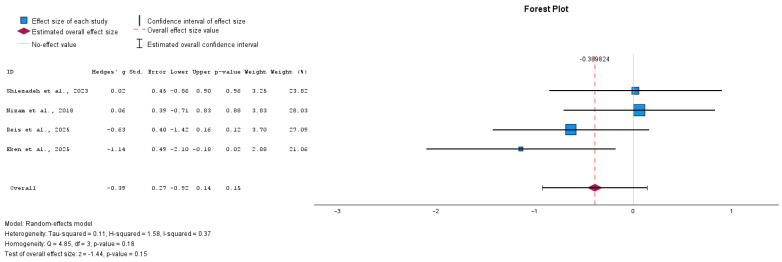
Forest plot of the effect of PRF on radiographic bone height following sinus augmentation [25,26,27,28].

Figure 6 shows the pooled analysis of radiographic bone height changes. The overall effect size was −0.39 (95% CI: −0.92 to 0.14), and the difference was not statistically significant (Z = −1.44, *p* = 0.15). Heterogeneity was moderate ($I^{2}=37\%)$. These findings suggest that, despite its positive effect on histological bone formation, PRF does not significantly improve radiographic bone height following sinus augmentation procedures.

Collectively, the meta-analysis demonstrates that PRF significantly improves histomorphometric outcomes, particularly by increasing newly formed bone and reducing residual graft material. However, no statistically significant effects were observed for connective tissue/marrow spaces or radiographic bone height. These findings suggest that PRF primarily enhances early biological healing and graft remodelling rather than producing consistent changes in radiographic bone dimensions.

## 4. Discussion

This systematic review aimed to evaluate and compare the histomorphometric outcomes of the regenerated vital bone and volume of new bone in the grafted maxillary sinus using allografts with and without PRF and xenografts with and without PRF in randomized clinical studies.

Overall, the available evidence suggests that the adjunctive use of PRF may contribute to bone regeneration primarily by promoting early osteogenesis and facilitating graft remodelling, particularly by increasing the proportion of newly formed bone and, in some cases, reducing the amount of residual graft material [30].

In the studies by Reis et al. and Shiezadeh et al., the addition of PRF was associated with higher percentages of newly formed bone and reduced residual graft material compared with graft materials used alone, indicating enhanced graft remodelling [25,27]. Similarly, Eken et al. reported a greater portion of newly formed bone in the T-PRF group compared to the DBBM-alone group after six months of healing [28]. In contrast, the split-mouth randomized clinical trial conducted by Nizam et al. demonstrated comparable levels of newly formed bone between combined PRF and control groups, although a lower percentage of residual graft particles was observed in the PRF group [26]. Collectively, these findings suggest that PRF may positively influence certain histological aspects of bone regeneration, particularly during the early stages of graft remodelling.

It should be noted that the included studies employed different PRF preparation protocols—L-PRF (Nizam et al.), H-PRF (Reis et al.), and T-PRF (Eken et al.), which differ in centrifugation speed, duration, and tube material and, consequently, in their cellular and structural composition. H-PRF is produced using horizontal centrifugation, which results in a smoother, more evenly distributed cell layer compared with L-PRF, while T-PRF is prepared in titanium tubes and, although structurally similar to L-PRF in platelet and leukocyte content, exhibits a thicker and denser fibrin architecture [17]. These differences in fibrin density and cellular distribution may influence the rate and magnitude of growth factor release and, therefore, the histomorphometric outcomes observed across studies. Pooling these distinct PRF subtypes in a single meta-analysis should therefore be interpreted as a limitation, as variability in PRF protocol may itself contribute to the moderate heterogeneity observed in some outcomes.

Radiographic findings across the included studies demonstrated largely comparable bone dimensions between PRF-treated and control groups. In the studies by Shiezadeh et al. and Nizam et al., no statistically significant differences were observed in augmented bone height or sinus dimensions following sinus augmentation procedures [25,26]. Similarly, Reis et al. reported comparable residual bone height between groups, although micro-CT analysis demonstrated improved bone microarchitecture in the PRF-treated sites. In contrast, Eken et al. observed greater radiographic bone density and graft volume in the DBBM group compared with the T-PRF group. These findings suggest that while PRF may enhance bone regeneration and graft remodelling, its influence on radiographic bone dimensions appears limited.

Developed by Dohan et al., as a second-generation platelet concentrate, PRF was subsequently incorporated into maxillary sinus augmentation protocols in 2006 [31]. Since its introduction, considerable research has focused on the biological properties of PRF, particularly the release of anti-inflammatory cytokines during fibrin matrix remodelling, indicating its healing properties [31]. The growth factors released during the first week following PRF preparation contribute to tissue maturation over the subsequent weeks. PRF has been extensively investigated as a biologic adjunct in regenerative procedures because of its ability to provide a sustained release of growth factors and cytokines that support tissue healing and bone regeneration [31,32]. Experimental studies have demonstrated that PRF may enhance osteoblast proliferation and differentiation while also serving as a fibrin matrix that facilitates wound healing and graft maturation [30,32,33].

From a clinical perspective, PRF has gained increased attention as a biological adjunct in maxillary sinus augmentation due to its favourable biological properties and ease of clinical application. The preparation of PRF does not require the use of anticoagulants or biochemical additives, making it a quite simple and cost-effective procedure [34]. In recent clinical and experimental studies, the use of PRF appeared to enhance early bone healing and improve the biological environment for implant placement following sinus augmentation procedures [19,35].

The findings of the present meta-analysis demonstrated a statistically significant improvement in histomorphometric outcomes favouring the use of PRF compared with graft materials alone (*p* = 0.01). The low heterogeneity observed among the included studies ($I^{2}$ = 17%) indicated a relatively high level of consistency in the reported effects. It should be noted, however, that with only four included studies, the statistical power to detect heterogeneity is limited, and a low $I^{2}$ value in this context should not be interpreted as definitive evidence of true homogeneity across studies.

These results suggest that PRF may positively influence early bone regeneration and graft remodelling. However, despite these statistically significant histological improvements, the overall clinical relevance should be interpreted with caution due to the limited number of included studies and variability in study protocols.

The present findings contrast with several prior meta-analyses reporting no significant benefit of PRF in sinus augmentation. One meta-analysis found no significant differences in newly formed bone, residual grafts, or soft-tissue area between the PRF and control groups [20], and a separate systematic review of PRF combined with bovine xenografts similarly reported no significant histomorphometric or radiographic benefit [36]. These discrepancies likely reflect differences in PRF protocols, graft materials, and studies included across reviews, underscoring that the evidence base for PRF in sinus augmentation remains mixed rather than uniformly favourable.

Although the meta-analysis demonstrated a statistically significant effect of PRF on newly formed bone (Hedges’ g = 0.61) and residual graft material (Hedges’ g = −0.65), the clinical significance of these findings warrants careful consideration. Hedges’ g values in this range are generally classified as small-to-moderate effect sizes, and the absolute differences in newly formed bone between PRF and control groups across the included studies ranged from approximately 0.1% (Nizam et al.) to 11.2% (Eken et al.). Whether an increase in this magnitude meaningfully improves implant primary stability, osseointegration, or long-term survival has not been directly established by the included studies, none of which reported implant-level outcomes such as insertion torque, implant stability quotient, or survival rates. Therefore, while the histological evidence supports a biologically favourable effect of PRF on early graft remodelling, translating this into a clinically meaningful advantage during implant placement and long-term rehabilitation remains to be confirmed by future studies incorporating implant-related clinical endpoints.

While these findings relate to early histological outcomes, long-term graft stability and peri-implant bone maintenance remain important clinical considerations. Giordano et al. reported that bone gain after maxillary sinus lift remained largely stable over a 5-year follow-up [37], while Trombelli et al. identified multiple factors influencing long-term peri-implant marginal bone resorption beyond the initial grafting procedure [38]. Whether PRF-enhanced early bone formation translates into improved long-term graft stability has not yet been directly investigated and warrants future study.

This discrepancy likely reflects differences in what each method measures: histomorphometry captures microscopic bone composition and maturation, whereas radiography measures macroscopic dimensional parameters that cannot resolve bone quality or remodelling stage. PRF may therefore accelerate early cellular bone formation without producing a detectable change in overall augmented bone volume within the short follow-up periods (four to six months) of the included studies. Longer-term studies combining both assessments would help clarify whether early histological changes eventually translate radiographically.

A primary limitation of this meta-analysis is the low number of included studies (k = 4), which inherently limits the precision and robustness of the pooled effect estimates and should be considered when interpreting the statistically significant finding reported above.

A further limitation of this meta-analysis relates to the clinical heterogeneity among the included studies. The four trials differed in the type of graft material used—allograft in Shiezadeh et al. and DBBM in Nizam et al., Reis et al., and Eken et al. These materials differ intrinsically in resorption rate, osteoconductivity, and remodelling behaviour independently of any PRF effect. Because each PRF condition was tested against a different graft material across studies, graft type and PRF use are not fully disentangled in the pooled analysis; consequently, the observed pooled effect size should be interpreted as reflecting PRF’s effect within the context of varying graft materials rather than an isolated, graft-independent effect of PRF itself. Healing periods also ranged from four to six months, and histomorphometric protocols varied slightly in biopsy site and parameters reported. While the low heterogeneity ($I^{2}$ = 17% for newly formed bone) suggests reasonably consistent effect directions, these differences should be considered when interpreting the pooled estimates. A formal assessment of publication bias (e.g., funnel plot asymmetry or Egger’s test) was not performed, as such methods require a substantially larger number of studies than the four included here to yield reliable results. The small number of included studies, therefore, represents an additional limitation, as the possibility of unpublished or unidentified studies with null or negative findings cannot be excluded. Additionally, the restriction of eligibility to English-language publications may have introduced language bias, as relevant studies published in other languages could not be assessed for inclusion and may have altered the pooled estimates.

PRF has been widely used as an adjunctive biomaterial in combination with bone graft substitutes during MSA procedures. Although numerous experimental and clinical studies have investigated its regenerative potential, the available evidence remains heterogeneous, and its clinical benefits have not been consistently demonstrated.

Our aim was to evaluate whether maxillary sinus augmentation with the use of PRF in combination with bone substitutes significantly affected the histomorphometric outcome of regenerated vital bone and the volume of newly formed bone. The current evidence from randomized clinical trials indicates that the addition of PRF does not consistently result in significantly greater radiographic bone volume compared with conventional grafting approaches. Therefore, PRF may be considered a supportive biological adjunct that may enhance the early healing environment rather than a replacement for establishing grafting materials in maxillary sinus augmentation procedures.

## 5. Conclusions

This systematic review indicates that the use of PRF as an adjunct during maxillary sinus augmentation may promote early bone maturation and graft remodelling, as reflected by increased newly formed bone and reduced residual graft materials in the included histomorphometric studies. However, the present evidence is insufficient to support long-term improvements in bone regeneration, implant-related outcomes, or overall clinical efficacy, as none of the included studies reported implant-level or extended follow-up data. Histomorphometric findings from the included randomized clinical studies suggest that PRF may increase the proportion of newly formed bone and, in some cases, reduce the amount of residual graft material. However, the regenerative outcomes were not consistently superior across all studies. Radiographic findings also demonstrated largely comparable bone dimensions between PRF-treated and control groups. Therefore, while PRF appears to be a promising biological adjunct in sinus augmentation procedures, the current evidence does not demonstrate consistently superior clinical outcomes across all grafting materials. The magnitude and consistency of these effects varied among the included studies, suggesting that the regenerative potential of PRF may be influenced by factors such as the type of graft material used, variations in PRF preparation protocols, and differences in healing periods [39].

The limitations of the included studies highlight the need for more robust randomized clinical trials with standardized PRF protocols and larger sample sizes to clarify their clinical effectiveness and long-term benefits in maxillary sinus augmentation.

## Figures and Tables

**Table 1:** Summary of the primary and secondary outcomes presented in the studies incorporated in the systematic review.

Author/Year	Design/Sample	Comparison	Follow-Up	Newly Formed Bone %/mm	Residual Graft %/mm	Marrow Generation (MG)/Connective Tissue (CT) %/mm	Radiographic Sinus Bone (Height (H)/Volume (V)/Density (D)/Width (W)%/mm)	Conclusion (Authors)
Shiezadeh et al., 2023 [25]	RCT; *n* = 20 patients	Group A: PRF + allograft; Group B: allograft	6 months	43.25 ± 5.22% (A) and 38.25 ± 7.01% (B).	9.35 ± 3.43% (A) and 13.18 ± 3.67% (B).	MG: 6.81 ± 2.19% (A) and 10.23 ± 4.49% (B).	W: 6.24 ± 1.07 mm (A) and 6.25 ± 1.27 mm (B). H: 2.74 ± 0.88 (A) and 2.72 ± 0.92 (B).	Addition of PRF to the allograft led to considerably lower bone marrow formation and lower residual graft material, while newly formed bone and radiographic bone dimensions were not statistically significant.
Nizam et al., 2018 [26]	Split-mouth RCT; *n* = 13 patients (26 sinuses)	DBBM (control) vs. DBBM + L-PRF (test)	6 months	21.38 ± 8.78% (test) and 21.25 ± 5.59% (control).	25.95 ± 9.54% (test) and 32.79 ± 5.89% (control).	CT: 52.67 ± 12.53% (test) and 45.96 ± 8.36% (control).	H (after 6 m.):13.60 ± 1.09 (test) and 13.53 ± 1.20 (control).	After 6 months of healing, the addition of L-PRF combined with DBBM did not significantly influence newly formed bone, residual graft percentage, connective tissue component, or radiographic bone height compared with DBBM alone.
Reis et al., 2025 [27]	RCT; *n* = 13 patients	DBBM (control) vs. DBBM + H-PRF (test)	4 months	51.33% ± 6.17% (test) and 45.68% ± 6.65% (control).	21.85% ± 8.93% (test) and 24.09% ± 11.79% (control).	CT: 26.81% ± 9.08% (test) and 30.21% ± 9.06% (control).	V: 30.38% ± 11.24% (test) and 21.38% ± 9.83% (control).	Compared with DBBM alone, maxillary sinuses augmented with H-PRF combined with DBBM demonstrated greater newly formed bone and bone volume compared with DBBM alone after 4 months of healing, while residual graft and connective tissue percentages remained comparable between the two groups.
Eken et al., 2025 [28]	Split-mouth RCT; *n* = 10 patients	T-PRF (Group A) vs. DBBM (Group B)	6 months	19.48 ± 14.60% (A) and 8.31 ± 5.47% (B).	0.00 ± 0.00% (A) and 4.81 ± 6.67% (B).	CT: 21.60 ± 12.43% (A) and 21.49 ± 11.77% (B).	H: 10.64 ± 3.96 mm (A) and 14.25 ± 1.65 mm (B).	Both T-PRF and DBBM yielded comparable histomorphometric results. Although DBBM showed greater radiographic bone height, no statistically significant differences were observed in newly formed bone, residual graft material, or connective tissue proportions.

Abbreviations: RCT—randomized clinical trial; DBBM—deproteinized bovine bone mineral; L-PRF—leukocyte and platelet-rich fibrin; H-PRF—platelet-rich fibrin obtained by horizontal centrifugation; T-PRF—titanium-prepared platelet-rich fibrin; MG—marrow generation; CT—connective tissue; H—height; V—volume; D—density; W—width.

## Data Availability

The original contributions presented in this study are included in the article. Further inquiries can be directed to the corresponding author.

## Appendix A

**Table A1:** PRISMA checklist.

Section and Topic	Item #	Checklist Item	Location Where Item Is Reported
**TITLE**			
Title	1	Identify the report as a systematic review.	Page 1
**ABSTRACT**			
Abstract	2	See the PRISMA 2020 for Abstracts checklist.	Page 1
**INTRODUCTION**			
Rationale	3	Describe the rationale for the review in the context of existing knowledge.	Pages 2–3
Objectives	4	Provide an explicit statement of the objective(s) or question(s) the review addresses.	Page 3
**METHODS**			
Eligibility criteria	5	Specify the inclusion and exclusion criteria for the review and how studies were grouped for the syntheses.	Pages 3–4
Information sources	6	Specify all databases, registers, websites, organisations, reference lists and other sources searched or consulted to identify studies. Specify the date when each source was last searched or consulted.	Page 3
Search strategy	7	Present the full search strategies for all databases, registers and websites, including any filters and limits used.	Page 3
Selection process	8	Specify the methods used to decide whether a study met the inclusion criteria of the review, including how many reviewers screened each record and each report retrieved, whether they worked independently, and if applicable, details of automation tools used in the process.	Pages 3–4
Data collection process	9	Specify the methods used to collect data from reports, including how many reviewers collected data from each report, whether they worked independently, any processes for obtaining or confirming data from study investigators, and if applicable, details of automation tools used in the process.	Pages 3–4
Data items	10a	List and define all outcomes for which data were sought. Specify whether all results that were compatible with each outcome domain in each study were sought (e.g., for all measures, time points, analyses), and if not, the methods used to decide which results to collect.	Pages 3–4
	10b	List and define all other variables for which data were sought (e.g., participant and intervention characteristics, funding sources). Describe any assumptions made about any missing or unclear information.	Pages 3–4
Study risk of bias assessment	11	Specify the methods used to assess risk of bias in the included studies, including details of the tool(s) used, how many reviewers assessed each study and whether they worked independently, and if applicable, details of automation tools used in the process.	Pages 4–5
Effect measures	12	Specify for each outcome the effect measure(s) (e.g., risk ratio, mean difference) used in the synthesis or presentation of results.	Page 4
Synthesis methods	13a	Describe the processes used to decide which studies were eligible for each synthesis (e.g., tabulating the study intervention characteristics and comparing against the planned groups for each synthesis (item #5)).	Page 3–4
	13b	Describe any methods required to prepare the data for presentation or synthesis, such as handling of missing summary statistics, or data conversions.	Page 4
	13c	Describe any methods used to tabulate or visually display results of individual studies and syntheses.	Page 4, 8–10
	13d	Describe any methods used to synthesize results and provide a rationale for the choice(s). If meta-analysis was performed, describe the model(s), method(s) to identify the presence and extent of statistical heterogeneity, and software package(s) used.	Page 4
	13e	Describe any methods used to explore possible causes of heterogeneity among study results (e.g., subgroup analysis, meta-regression).	Not reported
	13f	Describe any sensitivity analyses conducted to assess robustness of the synthesized results.	Not reported
Reporting bias assessment	14	Describe any methods used to assess risk of bias due to missing results in a synthesis (arising from reporting biases).	Not reported
Certainty assessment	15	Describe any methods used to assess certainty (or confidence) in the body of evidence for an outcome.	Not reported
**RESULTS**			
Study selection	16a	Describe the results of the search and selection process, from the number of records identified in the search to the number of studies included in the review, ideally using a flow diagram.	Pages 4–5
	16b	Cite studies that might appear to meet the inclusion criteria, but which were excluded, and explain why they were excluded.	Pages 4–5
Study characteristics	17	Cite each included study and present its characteristics.	Pages 5–6, Table 1
Risk of bias in studies	18	Present assessments of risk of bias for each included study.	Pages 5–6
Results of individual studies	19	For all outcomes, present, for each study: (a) summary statistics for each group (where appropriate) and (b) an effect estimate and its precision (e.g., confidence/credible interval), ideally using structured tables or plots.	Pages 10-11, Figure 3, Figure 4, Figure 5 and Figure 6
Results of syntheses	20a	For each synthesis, briefly summarise the characteristics and risk of bias among contributing studies.	Pages 5–6
	20b	Present results of all statistical syntheses conducted. If meta-analysis was done, present for each the summary estimate and its precision (e.g., confidence/credible interval) and measures of statistical heterogeneity. If comparing groups, describe the direction of the effect.	Pages 8–10
	20c	Present results of all investigations of possible causes of heterogeneity among study results.	Not reported
	20d	Present results of all sensitivity analyses conducted to assess the robustness of the synthesized results.	Not reported
Reporting biases	21	Present assessments of risk of bias due to missing results (arising from reporting biases) for each synthesis assessed.	Not reported
Certainty of evidence	22	Present assessments of certainty (or confidence) in the body of evidence for each outcome assessed.	Not reported
**DISCUSSION**			
Discussion	23a	Provide a general interpretation of the results in the context of other evidence.	Pages 10–11
	23b	Discuss any limitations of the evidence included in the review.	Pages 11–12
	23c	Discuss any limitations of the review processes used.	Pages 11–12
	23d	Discuss implications of the results for practice, policy, and future research.	Pages 11–12
**OTHER INFORMATION**			
Registration and protocol	24a	Provide registration information for the review, including register name and registration number, or state that the review was not registered.	Page 3
	24b	Indicate where the review protocol can be accessed, or state that a protocol was not prepared.	Not reported
	24c	Describe and explain any amendments to information provided at registration or in the protocol.	Not reported
Support	25	Describe sources of financial or non-financial support for the review, and the role of the funders or sponsors in the review.	Page 12
Competing interests	26	Declare any competing interests of review authors.	Page 12
Availability of data, code and other materials	27	Report which of the following are publicly available and where they can be found: template data collection forms; data extracted from included studies; data used for all analyses; analytic code; any other materials used in the review.	Not publicly available
